# Burden and Economic Impact of Antimicrobial Resistance in *Acinetobacter baumannii* in Iran: A 2000–2021 Analysis

**DOI:** 10.1155/cjid/6786134

**Published:** 2026-08-02

**Authors:** Sina Golestani, Ali Golestani, Samaneh Akbarpour, Mohammadreza Salehi, Arash Seifi, Keyhan Mohammadi, Maryam Shafaati

**Affiliations:** ^1^ Non-Communicable Diseases Research Center, Endocrinology and Metabolism Population Sciences Institute, Tehran University of Medical Sciences, Tehran, Iran, tums.ac.ir; ^2^ Endocrinology and Metabolism Research Center, Endocrinology and Metabolism Clinical Sciences Institute, Tehran University of Medical Sciences, Tehran, Iran, tums.ac.ir; ^3^ Sleep Breathing Disorders Research Center, Tehran University of Medical Sciences, Tehran, Iran, tums.ac.ir; ^4^ Infectious Diseases Department, Research Center for Antibiotic Stewardship and Antimicrobial Resistance, Imam Khomeini Hospital Complex, Tehran University of Medical Sciences, Tehran, Iran, tums.ac.ir; ^5^ Center for Communicable Disease Control (CDC), Ministry of Health and Medical Education, Tehran, Iran, behdasht.gov.ir; ^6^ Department of Clinical Pharmacy, School of Pharmacy, Tehran University of Medical Sciences, Tehran, Iran, tums.ac.ir

**Keywords:** *Acinetobacter baumannii*, antimicrobial resistance, burden of disease, economic burden, Iran

## Abstract

**Background:**

*Acinetobacter baumannii* is a major contributor to antimicrobial resistance (AMR), particularly in hospitals. Its rising resistance presents significant clinical and economic concerns. This study evaluated the national burden and economic impact of *A*. *baumannii* infections in Iran from 2000 to 2021 using GBD 2021 AMR data.

**Materials and Methods:**

We estimated mortality and disability‐adjusted life years (DALYs) per 100,000 people with 95% UIs under two scenarios: resistant infections replaced by susceptible ones (attributable burden) and replaced by no infection (associated burden). Economic burden was calculated using GDP per capita and purchasing power parity (PPP)–adjusted estimates.

**Results:**

From 2000 to 2021, total associated DALYs declined from 90,391.9 (95% UI: 80,178.0–100,605.9) to 69,740.5 (65,132.4–74,348.6) and attributable DALYs from 35,284.5 (30,843.1–39,725.9) to 28,085.9 (25,415.9–30,755.9). However, associated deaths increased from 2019.5 (1870.3–2168.8) to 2523.7 (2322.5–2724.9), with neonates and individuals aged 50 years and older most affected. Bloodstream and lower respiratory infections contributed most to DALYs. Resistance remained highest for cephalosporins and carbapenems. The economic burden peaked in 2010 at 584.6 million PPP and decreased to 435.2 million in 2021.

**Conclusion:**

Although disease burden declined, rising mortality and resistance trends highlight the need for stronger infection control and stewardship to reduce *A. baumannii*‐related AMR in Iran.

**Highlights**
• Total DALYs from *Acinetobacter baumannii* declined from 90,000 in 2000 to 69,000 in 2021.
• Attributable DALYs fell, but overall deaths rose, especially in neonates and the elderly.
• Carbapenem and cephalosporin resistance remained highest among drug classes.
• Bloodstream and respiratory infections led to DALY and mortality contributions.
• Economic burden peaked at 584 million PPP USD in 2010, decreasing to 435 million by 2021.

## 1. Introduction

Antimicrobial resistance (AMR) is one of the most pressing threats to global public health today. AMR occurs when microorganisms, such as bacteria, become resistant to antibiotics, rendering standard treatments ineffective and allowing infections to persist. Each year, bacterial infections contribute to approximately 7.7 million deaths worldwide, with 1.27 million of these directly attributed to AMR [1]. Projections suggest that if current trends continue, AMR could be responsible for 10 million deaths annually by 2050 [2]. Recognizing the severity of the issue, the World Health Organization (WHO) identified “combatting drug resistance” as one of the ten critical global health challenges to be tracked in 2021 [3].

In Iran, AMR poses a significant public health challenge. Studies indicate that AMR rates are not only increasing nationwide but are particularly higher in the northern and southern regions of the country [4–6]. Ensuring appropriate access to antibiotics is crucial for improving life expectancy and reducing healthcare costs. However, AMR may threaten these benefits. Additionally, AMR serves as a barrier to achieving certain Sustainable Development Goals (SDGs), particularly those aimed at reducing preventable deaths among newborns and vulnerable populations [7].

Among the most concerning drug‐resistant pathogens is *Acinetobacter baumannii*, a member of the ESKAPE group—comprising *Enterococcus faecium, Staphylococcus aureus, Klebsiella pneumoniae, A. baumannii, Pseudomonas aeruginosa, and Enterobacter* spp.—which are classified as critical multidrug‐resistant (MDR) organisms posing major challenges to healthcare systems [8]. *A. baumannii* is particularly problematic due to its resistance to last‐resort antibiotics such as carbapenems, leading the Centers for Disease Control and Prevention (CDC) to categorize it as a significant public health threat [9, 10]. Reports from various regions indicate that MDR *A. baumannii* has been responsible for outbreaks worldwide [11]. In Iran, *Acinetobacter* spp. have been identified as the most prevalent pathogen in healthcare‐associated infections, with a reported prevalence of 25%, contributing to increased mortality rates [12]. Studies have highlighted several risk factors associated with MDR *A. baumannii*, including male sex, cardiovascular disease, and prior antibiotic use [13].

While aggregate global and regional reports from broad AMR initiatives provide essential macro‐level trends, they frequently obscure the highly specific trajectories of individual high‐threat pathogens within localized healthcare systems. A pathogen‐specific analysis of *A. baumannii* is uniquely warranted in Iran, where this organism has emerged as a leading cause of severe, highly resistant healthcare‐associated infections. Furthermore, macro‐level global data lack localized economic context. To address this gap, this study pairs health metrics from the IHME MICROBE platform, leveraging data from the Global Burden of Disease (GBD) 2021 study across 204 countries and territories [14], with our own country‐specific economic modeling. By doing so, we provide a long‐term national evaluation of both the clinical and financial toll of *A. baumannii* in Iran from 2000 to 2021. Ultimately, these targeted insights offer Iranian public health authorities the precise baseline evidence required to tailor national antimicrobial stewardship action plans and strategically allocate healthcare budgets.

## 2. Materials and Methods

### 2.1. Overview

The research utilized AMR estimates from the Institute for Health Metrics and Evaluation’s (IHME) 2021 global assessment. These estimates combined mortality and disease incidence data from the GBD study with multiple epidemiological sources to evaluate resistance effects across 11 infection types, 22 bacterial species, 16 antibiotic classes, and 84 specific pathogen–drug interactions in 204 geographical regions. Full methodological details were previously documented [15, 16]. For this investigation, *A. baumannii* resistance patterns in Iran were extracted from IHME’s MICROBE platform [17]. The study complied with GATHER guidelines for transparent health metric reporting [18, 19]. We performed original analyses in this study, including economic burden estimation. All other results are visualizations or tables derived from the public IHME MICROBE database [17].

### 2.2. Data Sources

The AMR project aggregated diverse data inputs to assess resistance burden, including death certificates, hospital admissions (inpatient and outpatient), insurance claims, microbiological laboratory records (both linked and unlinked to clinical outcomes), peer‐reviewed studies, single‐drug resistance profiles, antibiotic consumption in pediatric populations, and mortality surveillance systems. GBD cause‐of‐death estimates were also incorporated into the modeling framework. In total, more than 520 million data points spanning 19,513 location‐years were evaluated. Further information on data selection and processing can be found in prior publications [15].

### 2.3. Data Processing and Modelling

A 10‐stage analytical framework was employed to estimate AMR burden. First, sepsis‐related fatalities were identified using GBD 2021 mortality data, and these deaths were then classified into 22 infectious syndromes via mixed‐effects logistic regression—11 of which had sufficient data for pathogen‐specific AMR assessment. Subsequent steps involved modeling pathogen‐specific case fatality rates (CFRs) with the RegMod tool and determining pathogen distribution across syndromes using over 24 million records of microbial isolates. A multinomial model adjusted for disparities between data sources and covariates such as the Healthcare Access and Quality (HAQ) Index.

Steps 5–7 estimated resistance prevalence for 84 key pathogen–drug combinations using a two‐stage spatiotemporal model, correcting for laboratory interpretation discrepancies and sampling biases. National antibiotic consumption data were integrated to refine model precision. A novel optimization technique was applied to assess multidrug resistance by estimating composite resistance patterns.

Steps 8–9 computed the relative mortality risk and prolonged hospitalization associated with resistant versus susceptible infections, employing meta‐regression to derive population attributable fractions (PAFs). The final step (Step 10) estimated AMR burden under two counterfactual scenarios: (A) resistant infections replaced by drug‐susceptible ones (attributable burden) and (B) resistant infections replaced by no infection (associated burden).

### 2.4. Health Burden Measures

The analysis reported deaths and disability‐adjusted life years (DALYs) per 100,000 population attributable to *A. baumannii* resistance, differentiating between associated and attributable burdens. Resistance‐attributable deaths were calculated by multiplying syndrome‐specific mortality, sepsis‐related death proportions, pathogen‐specific syndrome distributions, and mortality PAFs for each resistance profile. Years of Life Lost (YLLs) were derived using standard GBD methodologies, incorporating age‐stratified life expectancy tables [19].

Years Lived with Disability (YLDs) were estimated by combining infection incidence rates, pathogen‐specific etiology shares, per‐case YLDs, and nonfatal outcome PAFs. For MDR cases, burdens were proportionally allocated to prevent overlap, ensuring unique estimates for each pathogen–drug pair. DALYs were the sum of YLLs and YLDs. The total AMR burden under the drug‐sensitive counterfactual was obtained by aggregating all pathogen–drug combinations. A parallel approach estimated associated burden, substituting mortality PAFs with resistance prevalence in fatalities.

### 2.5. Economic Burden

Following Copenhagen Consensus guidelines [20], economic losses associated with *A. baumannii* AMR were estimated by assigning a monetary value to DALYs using one‐ and three‐times GDP per capita [21]. GDP in linked current local currency units was adjusted to 2021 prices using the World Bank linked‐series GDP deflator and converted to U.S. dollars using Iran’s 2021 market exchange rate [22]. These estimates are, therefore, expressed in constant 2021 U.S. dollars. For cross‐national comparability, annual GDP reported by the World Bank in constant 2021 international dollars (purchasing power parity [PPP]) was divided by the corresponding annual GBD population estimate [22]. Results were reported under four valuation scenarios: 1× and 3× GDP per capita in constant 2021 U.S. dollars and 1× and 3× GDP per capita in constant 2021 international dollars.

### 2.6. Statistical Analysis

Uncertainty was propagated throughout the analysis by generating 100 posterior draws for each age–sex–year–location stratum. These draws were used to compute final estimates for resistance‐related and resistance‐attributable deaths and infections. Ninety‐five percent uncertainty intervals (95% UIs) were derived as ±1.96 standard deviations from the mean [15]. Python (v3.12.4) was used for statistical analysis and visualization. Statistical significance was assessed based on the nonoverlap of the UIs.

## 3. Results

### 3.1. Trends in Burden and Mortality

**FIGURE 1:**
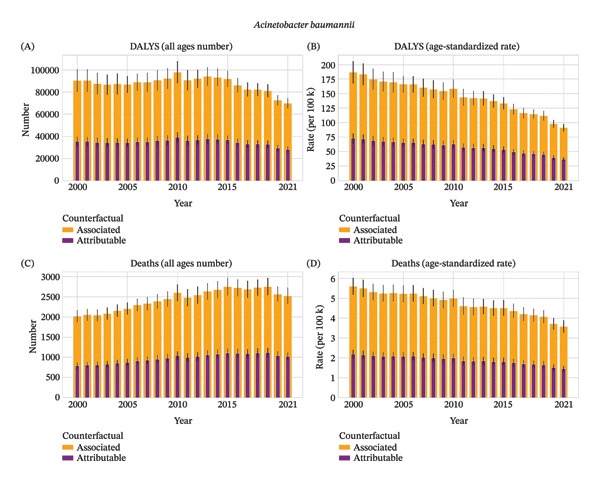
Trends of associated and attributable numbers and rates of burden and mortality caused by *Acinetobacter baumannii* in Iran from 2000 to 2021. DALYs = disability‐adjusted life years.

Between 2000 and 2021, the trends in both the number and age‐standardized rate of DALYs and deaths caused by *A. baumannii* were analyzed in Iran (Figure 1). The patterns of both associated and attributable burdens followed similar trajectories over the two decades.

The total number of associated DALYs experienced a slight significant decline, decreasing from 90,391.9 (95% UI: 80,178.0–100,605.9) in 2000 to 69,740.5 (65,132.4–74,348.6) in 2021. Similarly, the attributable DALYs significantly decreased from 35,284.5 (30,843.1–39,725.9) in 2000 to 28,085.9 (25,415.9–30,755.9) in 2021. A more pronounced decline was observed in the DALY rate, with the associated rate per 100,000 population significantly dropping from 186.8 (167.5–206.0) in 2000 to 91.2 (84.9–97.6) in 2021. The attributable DALY rate followed a similar trend, significantly decreasing from 72.9 (64.3–81.5) per 100,000 population in 2000 to 36.7 (33.2–40.3) in 2021.

In contrast to DALYs, mortality due to *A. baumannii* showed an increase in absolute numbers, though the rate per 100,000 population declined over time. The number of associated deaths significantly rose from 2019.5 (95% UI: 1870.3–2168.8) in 2000 to 2523.7 (2322.5–2724.9) in 2021, while the attributable deaths significantly increased from 789.2 (710.7–867.8) in 2000 to 1019.7 (916.9–1122.4) in 2021. However, despite the increase in death counts, the associated death rate per 100,000 population significantly declined from 5.6 (5.2–6.0) in 2000 to 3.6 (3.3–3.9) in 2021. A similar trend was seen in the attributable death rate, which significantly fell from 2.2 (2.0–2.4) per 100,000 population in 2000 to 1.4 (1.3–1.6) in 2021.

Furthermore, the burden and mortality caused by *A. baumannii* infections among susceptible cases were analyzed in Iran for the years 2000 and 2021 (Supporting File: Figure 1). Overall, both the number and rate of DALYs and deaths related to susceptibility exhibited a nonsignificant declining trend over these two decades. Additionally, the burden and mortality among susceptible cases were negligible compared to the total associated burden and mortality. Specifically, the total number of DALYs among susceptible individuals decreased from 2828.3 (95% UI: 315.5–5341.1) in 2000 to 1295.3 (43.0–2547.5) in 2021. Likewise, the DALY rate per 100,000 population dropped from 5.8 (0.7–11.0) to 1.7 (0.1–3.3). In terms of mortality, the number of deaths declined from 63.1 (7.0–119.3) in 2000 to 46.8 (1.1–92.4) in 2021, while the mortality rate decreased from 0.2 (0.0–0.3) to 0.1 (0.0–0.1) per 100,000 population.

### 3.2. Burden and Mortality Across Age Groups

**FIGURE 2:**
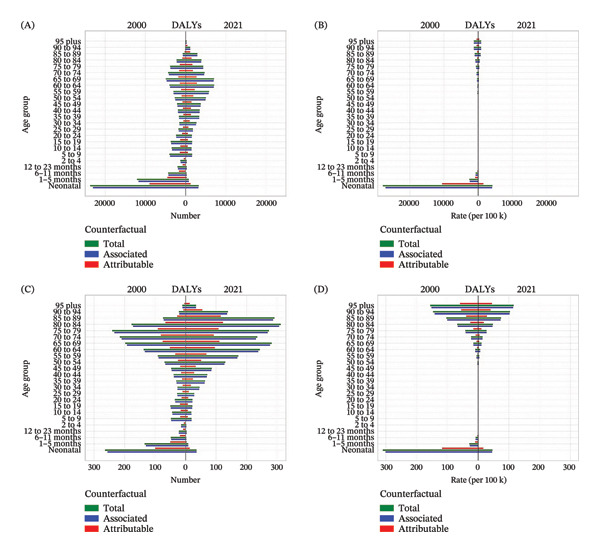
Burden and mortality of *Acinetobacter baumannii* across various age groups in Iran in 2000 and 2021. DALYs = disability‐adjusted life years.

The associated, attributable, and total number and rate of DALYs and deaths caused by *A. baumannii* were analyzed across different age groups in Iran for the years 2000 and 2021 (Figure 2).

In 2000, *A. baumannii* imposed the highest burden and mortality on the neonatal age group. The total number of DALYs in this group was 23,818.7 (95% UI: 16,398.7–31,238.8), with associated and attributable DALYs reaching 23,096.6 (15,859.6–30,333.6) and 9025.9 (6469.4–11,582.4), respectively. When considering DALY rates per 100,000 population, the neonatal group exhibited the highest values, with a total rate of 27,971.6 (19,257.8–36,685.4), an associated rate of 27,123.5 (18,624.7–35,622.3), and an attributable rate of 10,599.6 (7597.3–13,601.8). Regarding mortality, the neonatal group also had the highest death toll, with total deaths numbering 264.6 (182.2–347.1). The associated and attributable number of deaths were 256.6 (176.2–337.0) and 100.2 (71.9–128.7), respectively. Death rates per 100,000 population in this group were also high, with a total rate of 310.7 (213.9–407.6), an associated rate of 301.3 (206.9–395.8), and an attributable rate of 117.8 (84.4–151.1). Apart from neonates, the DALY burden was relatively similar across other age groups, whereas mortality rates showed an increase in individuals aged 50 years and older.

By 2021, the pattern of disease burden had shifted. Unlike in 2000, the neonatal age group no longer exhibited the highest mortality or disease burden. Instead, DALY numbers were more evenly distributed across age groups, with the 60‐ to 64‐year age group recording the highest total DALYs at 7231.3 (95% UI: 6120.5–8342.1). However, DALY rates per 100,000 population remained highest in the neonatal group, with a total rate of 4264.1 (2746.0–5782.2). In terms of mortality, older adults had the highest number of deaths, particularly the 80‐ to 84‐year age group, which recorded a total of 313.3 deaths (261.3–365.3). Death rates were also highest in the elderly and neonatal populations, with the 95‐plus age group had the highest total death rate at 116.4 (81.5–151.3) per 100,000 population.

### 3.3. Antibiotic Resistance Trends

**FIGURE 3:**
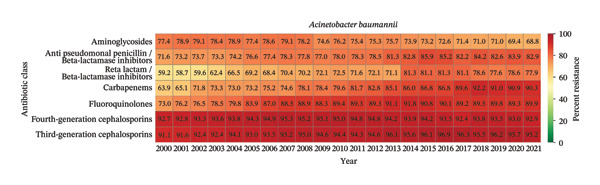
Antibiotic resistance trends of *Acinetobacter baumannii* in Iran (2000–2021).

The resistance percentages of *A. baumannii* to various antibiotic classes were analyzed from 2000 to 2021, revealing an overall increase in antibiotic resistance over the years (Figure 3). Among the studied antibiotic classes, carbapenem resistance exhibited the highest increase, rising from 63.9% in 2000 to 90.3% in 2021.

On the other hand, beta‐lactam/beta‐lactamase inhibitors initially exhibited the lowest resistance rate at 59.2% in 2000, but this steadily increased, reaching 77.9% in 2021. Similarly, resistance to antipseudomonal penicillins combined with beta‐lactamase inhibitors followed a rising trend, starting at 71.6% in 2000 and reaching 82.9% in 2021. Additionally, resistance to fluoroquinolones increased from 73.0% in 2000 to 89.9% in 2021.

The highest resistance rates were observed for third‐ and fourth‐generation cephalosporins, which were already above 90% at the beginning of the study period. Resistance to third‐generation cephalosporins increased from 91.1% in 2000 to 95.2% in 2021, while fourth‐generation cephalosporins showed a slight increase from 92.7% in 2000 to 92.9% in 2021.

Interestingly, aminoglycoside resistance demonstrated a decreasing trend, declining from 77.4% in 2000 to 68.8% in 2021. Among all antibiotic classes examined in 2021, aminoglycosides exhibited the lowest resistance levels.

### 3.4. The Attributable Burden and Mortality due to Antibiotic Resistance

**FIGURE 4:**
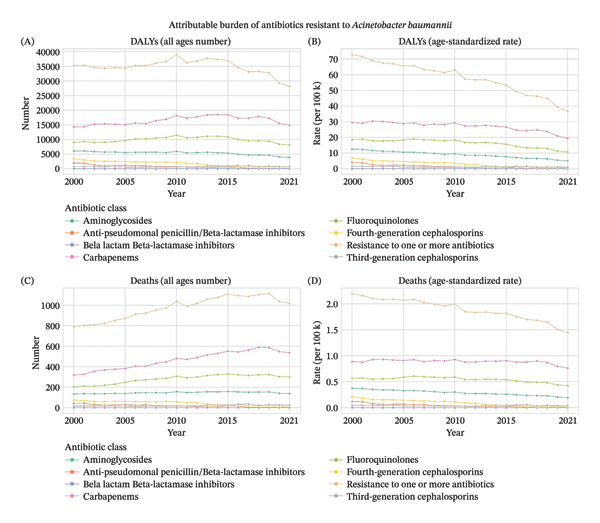
The attributable burden and mortality due to antibiotic resistance in *Acinetobacter baumannii* in Iran from 2000 to 2021. DALYs = disability‐adjusted life years.

The attributable burden and mortality due to antibiotic resistance in *A. baumannii* were analyzed in Iran from 2000 to 2021 (Figure 4). Over this two‐decade period, the trend of attributable DALYs for each antibiotic class showed a general decline. Overall, the total burden of antibiotic resistance in *A. baumannii* exhibited a decreasing trend. Specifically, the attributable DALYs associated with resistance to one or more antibiotics significantly declined from 35,284.5 (95% UI: 30,843.1–39,725.9) in 2000 to 28,085.9 (25,415.9–30,755.9) in 2021. Similarly, the DALY rate per 100,000 population significantly dropped from 72.9 (64.3–81.5) in 2000 to 36.7 (33.2–40.3) in 2021. When examining individual antibiotic classes, resistance to carbapenems consistently contributed the highest DALY burden, followed by fluoroquinolones and aminoglycosides. Other antibiotic classes, such as cephalosporins and beta‐lactam/beta‐lactamase inhibitors, had lower DALY numbers and rates throughout the study period.

Despite the observed reduction in DALYs, mortality due to antibiotic‐resistant *A. baumannii* displayed a contrasting trend. The number of deaths attributed to resistance increased slightly for most antibiotic classes. Overall, the total attributable deaths due to resistance to one or more antibiotics significantly rose from 789.2 (95% UI: 710.7–867.8) in 2000 to 1019.7 (916.9–1122.4) in 2021. However, the age‐standardized death rate per 100,000 population significantly decreased from 2.2 (2.0–2.4) in 2000 to 1.4 (1.3–1.6) in 2021. Among specific antibiotic classes, resistance to carbapenems was associated with the highest number of deaths. Resistance to fluoroquinolones and aminoglycosides also contributed substantially to mortality.

### 3.5. Burden and Mortality of Different Infectious Syndromes

**FIGURE 5:**
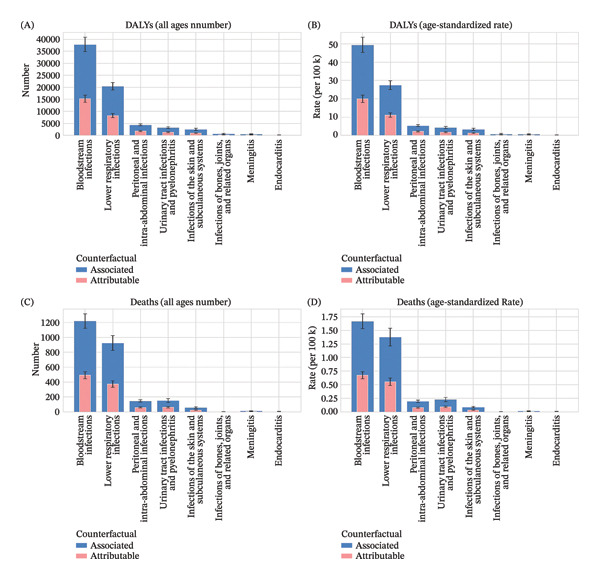
The Associated and attributable numbers and rates of burden and mortality of *Acinetobacter baumannii* infection syndromes in Iran, in 2021. Error bars represent 95% uncertainty intervals. DALYs = disability‐adjusted life years.

In 2021, the associated and attributable burden of *A. baumannii* infections in Iran was analyzed across different infectious syndromes, including both the total number and age‐standardized rates of DALYs and deaths (Figure 5). The highest burden was observed in bloodstream infections, followed by lower respiratory infections. In contrast, infections of the bones and joints, meningitis, and endocarditis had the lowest burden and mortality.

For bloodstream infections, the associated DALY burden was 37,868.9 (95% UI: 34,907.2–40,830.5), with an age‐standardized rate of 49.4 (45.3–53.5) per 100,000 population. The attributable DALY burden for bloodstream infections was 15,299.0 (13,776.0–16,822.0), with a corresponding rate of 20.0 (17.9–22.0) per 100,000 population.

A similar pattern was observed for mortality. The associated number of deaths due to bloodstream infections was 1219.3 (95% UI: 1122.7–1315.8), with an age‐standardized rate of 1.7 (1.5–1.8) per 100,000 population. The attributable deaths were 492.6 (444.8–540.5), with a rate of 0.7 (0.6–0.7) per 100,000 population.

A comparison of the associated and attributable burden and mortality of different infectious syndromes caused by *A. baumannii* between 2000 and 2021 revealed consistent patterns over time (Supporting File: Figure 2). Throughout this period, bloodstream infections and lower respiratory infections remained the primary contributors to the burden and mortality of *A. baumannii* infections.

### 3.6. Economic Burden

The economic burden associated with *A. baumannii* infections in Iran was analyzed for the years 2000–2021 (Supporting File: Figure 3). In the 1 × GDP per capita scenario, the associated economic burden was estimated at 286.6 (UI 95%: 254.2–319.0) million USD in 2000, peaked significantly at 425.8 (382.2–469.5) million USD in 2010, and declined significantly to 313.3 (292.6–334.0) million USD in 2021. Under the 3× GDP per capita scenario, the associated economic burden followed a similar trend, increasing significantly from 859.8 (762.6–956.9) million USD in 2000 to 1277.5 (1146.5–1408.5) million USD in 2010, before declining significantly to 939.9 (877.8–1002.0) million USD in 2021.

The attributable economic burden was 111.9 (UI95%: 97.8–126.0) million USD in 2000, rose significantly to 169.4 (149.6–189.4) million USD in 2010, and then decreased significantly to 126.2 (114.2–138.2) million USD in 2021 under the 1× scenario. In the 3× GDP per capita scenario, attributable costs were 335.6 (293.4–377.9) million USD in 2000, peaked significantly at 508.5 (448.7–568.3) million USD in 2010, and declined significantly to 378.5 (342.5–414.5) million USD in 2021.

When measured in PPP, the associated economic burden in the 1× scenario was 988.6 (UI95%: 876.9–1100.4) million PPP in 2000, peaked significantly at 1468.8 (1318.2–1619.4) million PPP in 2010, and then declined significantly to 1080.6 (1009.2–1152.0) million PPP in 2021. Under the 3× scenario, the corresponding estimates were 2965.7 (2630.6–3300.8) million PPP in 2000, increased significantly to 4406.3 (3954.5–4858.1) million PPP in 2010, and then declined significantly to 3241.9 (3027.7–3456.1) million PPP in 2021.

The attributable economic burden followed a similar trend. In the 1× scenario, costs were 385.9 (UI95%: 337.3–434.5) million PPP in 2000, peaked significantly at 584.6 (515.9–653.4) million PPP in 2010, and declined significantly to 435.2 (393.8–476.6) million PPP in 2021. Under the 3× scenario, attributable costs were 1157.6 (1011.9–1303.4) million PPP in 2000, increased significantly to 1753.9 (1547.7–1960.1) million PPP in 2010, and then declined significantly to 1305.6 (1181.4–1429.7) million PPP in 2021.

## 4. Discussion

To our knowledge, this study provides the most comprehensive analysis of AMR in *A. baumannii* in Iran to date. The findings indicate that while the burden and mortality rates associated with *A. baumannii* infections in Iran have slightly decreased over the past two decades, they remain a significant public health concern. Evidence from the WHO Eastern Mediterranean Region suggests that antimicrobial stewardship interventions, primarily through audit and feedback, are effective in reducing antibiotic resistance and usage [23, 24], while research in Iran specifically links infection prevention and control (IPC) measures to a decreased prevalence of carbapenem‐resistant *A. baumannii* [25]. Therefore, the modest decline in disease burden and mortality observed in this study may reflect gradual improvements in IPC and stewardship programs. However, the persistently high mortality underscores the opposing force of escalating multidrug and extensively drug‐resistant *A. baumannii* strains, which severely constrain therapeutic efficacy [26, 27]. Interestingly, while Iran has observed a slight decline in the burden, studies from other regions report an increasing trend in the prevalence and resistance of *A. baumannii* infections [28]. This divergence may reflect differences in regional implementation of antimicrobial stewardship policies and the effectiveness of IPC interventions [29–31]. It has also been established that the incidence and prevalence of *A. baumannii* infections in intensive care units (ICUs) are 10–50 times higher than those in general hospital settings. Additionally, *A. baumannii* is the most frequently identified pathogen among nosocomial infections in ICUs [32, 33], a trend that also holds true for Iran [12].

This study explored the relationship between age and the burden of *A. baumannii* infections. In 2000, neonates experienced the highest DALYs and death rates in Iran. However, by 2021, this burden had shifted primarily to older age groups. This epidemiological transition likely results from decreased bacterial resistance in neonatal intensive care units (NICUs) [34]. Concurrently, Iran’s population has aged significantly [35], which is a key risk factor for *A. baumannii* mortality. Other studies have also shown that *A. baumannii* infections are associated with increased mortality in elderly patients, likely due to reduced drug efficacy in this population. Several mechanisms have been proposed to explain this increased susceptibility, including an exaggerated inflammatory response leading to tissue destruction and a decline in myeloperoxidase levels, which impairs the bactericidal function of inflammatory cells [36, 37]. Interestingly, another study found that males are more susceptible to *A. baumannii* infections than females, a difference potentially attributed to the site of infection and variations in hormonal levels [38].

The resistance patterns of *A. baumannii* to various antibiotics were also analyzed. Overall, resistance rates have increased over time, with the highest resistance observed against cephalosporins. The most significant increase in resistance was observed for carbapenems, while aminoglycoside resistance exhibited a declining trend over the past two decades. A systematic review in Iran also revealed an increasing trend in AMR among *A. baumannii* strains [39]. A study in northwestern Iran reported that cephalosporin resistance was the most common form of resistance in *A. baumannii* infections [40]. Another study in western Iran documented the spread of resistance genes, leading to the emergence of AMR in this region [41]. Many studies worldwide corroborate the increasing trend of AMR in *A. baumannii*, particularly regarding carbapenem resistance [9, 11, 42–44]. The mechanisms underlying carbapenem resistance may involve modifications in porins, alterations in penicillin‐binding proteins, or the production of β‐lactamases [45–47]. Regarding cephalosporin resistance, some studies have identified an upregulation of the *ampC* gene and horizontal gene transfer as contributing factors [48, 49]. Notably, a study conducted in the United States found that MDR *A. baumannii* exhibited a decreasing trend, which was attributed to the implementation of antimicrobial stewardship programs [50]. The observed patterns of aminoglycoside resistance in *A*. *baumannii* clinical isolates in Iran are primarily mediated by a diverse array of aminoglycoside‐modifying enzymes (AMEs). The prevalence and distribution of these enzymes may explain the temporal shifts in resistance profiles for this antibiotic class [51].

During the study period, the overall burden and mortality rates associated with *A. baumannii* infections exhibited a declining trend. Among different antimicrobial‐resistant strains, carbapenem‐resistant *A. baumannii* was associated with the highest mortality rates. Similarly, previous studies have reported that carbapenem resistance in *A. baumannii* significantly increases patient burden and mortality compared to carbapenem‐susceptible *A. baumannii* infections [42, 52, 53]. A study on sepsis patients also revealed that MDR *A. baumannii* infections were associated with a higher mortality rate compared to non‐MDR infections [54]. Interestingly, some studies found no significant difference in mortality rates between AMR and non‐AMR *A. baumannii* infections in surgical ICUs [55]. This study identified bloodstream and lower respiratory infections as the main contributors to the burden and mortality of *A. baumannii* infections. Consistently, other studies have reported that ventilator‐associated pneumonia and bloodstream infections are the most common syndromes caused by *A. baumannii* [56].

Over the two decades covered by this study, the economic burden associated with *A. baumannii* infections initially increased between 2000 and 2010, followed by a decline in subsequent years. Previous studies have also highlighted the substantial financial impact of *A. baumannii* infections. One study found that patients with MDR *A. baumannii* infections incurred an additional hospitalization cost exceeding $1000 per admission [57]. Another study reported an excess patient charge of over $60,000 and an increased hospital stay of approximately 13 days [56]. In another study, the additional costs were more than 3500 dollars [54]. A systematic review revealed that the attributable cost of resistant infections was around 2000–30,000 U.S. dollars in different studies [58]. A comparative study showed that *A. baumannii* infections led to twice the hospitalization costs compared to non‐*A. baumannii* infections [59]. Furthermore, it has been estimated that preventing a carbapenem‐resistant *A. baumannii* outbreak could have saved approximately $300,000 [60]. To mitigate these financial burdens, several cost‐effective strategies have been suggested. One study proposed routine screening for *A. baumannii* in ICUs as a cost‐effective intervention [61]. Another study emphasized the benefits of whole‐genome sequencing and shotgun metagenomics in infection control, highlighting their potential to improve clinical outcomes while being economically viable [62]. The economic trend observed in our study underscores that while progress has been made, sustained funding for stewardship, surveillance, and innovative diagnostics is crucial to further reduce the financial impact of *A. baumannii* AMR.

The observed decline in the burden of *A. baumannii* infections in Iran can be partially attributed to the implementation of key national policies and surveillance systems. A cornerstone of this effort is the National Action Plan of the Islamic Republic of Iran for Combating Antimicrobial Resistance (NAP‐IRIAMR), aligned with the WHO Global Action Plan and active from 2016 to 2021. This strategic framework established five core objectives to address AMR: raising public awareness and education, strengthening AMR surveillance, preventing the spread of resistant organisms, promoting the rational use of antimicrobials, and fostering AMR‐related research and development. Notably, the NAP‐IRIAMR specifically identifies *Acinetobacter* species as a leading cause of healthcare‐associated infections, underscoring its priority status [63]. Complementing this policy framework, enhanced surveillance has provided critical data to guide interventions. The Iranian Nosocomial Infection Surveillance (INIS) system, a hospital‐based platform, has been instrumental in monitoring infection trends. Recent data from this system indicate that healthcare‐associated infections, particularly pneumonia, urinary tract infections, and surgical site infections, remain prevalent, with the highest incidence occurring in ICUs [64]. Empirical evidence confirms the effectiveness of focused interventions derived from these national strategies. Specific IPC measures have been shown to reduce rates of carbapenem‐resistant *A. baumannii* [65]. Furthermore, the establishment of a National Antimicrobial Stewardship Program (NASP) has successfully reduced overall antimicrobial consumption [66]. More targeted stewardship programs focusing on high‐priority agents like carbapenems have demonstrated success in reducing associated healthcare costs [67]. Collectively, these coordinated policy, surveillance, and intervention efforts represent a multifaceted approach that has contributed to mitigating the public health and economic impact of *A. baumannii* in Iran.

Despite increasing research on AMR in *A. baumannii* infections, further studies are needed to assess the burden and mortality rates across different regions. The trends observed in Iran, such as the shift in burden from neonates to the elderly and the initial rise followed by a decline in economic impact, may not be uniform across other settings. Variations in healthcare infrastructure, accessibility of diagnostics, antimicrobial consumption patterns, and the implementation timeline of national AMR action plans could lead to divergent epidemiological and economic trajectories. Each region employs distinct infection control strategies, and understanding these variations could help refine interventions and identify transferable best practices. Future research should therefore adopt a comparative approach, examining how differences in policy, stewardship, and demographic transitions influence the local burden of *A. baumannii* AMR to support more tailored and effective public health responses globally.

This study has several strengths. First, it utilizes data from the GBD 2021 study and the World Bank, two of the most comprehensive and up‐to‐date global databases. Second, it includes age‐specific analyses, providing valuable insights into population‐level susceptibility. Third, it offers a detailed examination of both the clinical and economic burden of AMR in *A. baumannii* infections in Iran over the past two decades. However, this study also has some limitations. Relying on model‐based estimates may introduce inaccuracies due to data gaps or biases in input sources. Additionally, since the study focuses exclusively on Iran, its findings may not be generalizable to other regions with different environmental conditions, an issue known as the ecological fallacy. Lastly, this study did not investigate the specific risk factors contributing to the burden of *A. baumannii* infections, which limits the mechanistic interpretation of the observed trends and should be addressed in future research. Further studies should also conduct provincial‐level analyses within Iran to better understand regional variations in AMR trends and their implications.

## 5. Conclusion

This study provides a comprehensive, two‐decade analysis of the evolving health and economic burden of *A. baumannii* infections in Iran. While overall DALY rates have declined, likely reflecting improvements in infection control and healthcare access, the rising absolute mortality and persistently high resistance to last‐resort antibiotics underscore an escalating crisis, particularly among the elderly and in hospital‐acquired bloodstream and respiratory infections. The divergence between declining burden rates and increasing death counts highlights the formidable challenge posed by demographic aging and the proliferation of MDR strains, which outpace current containment efforts.

Key recommendations emerge from these findings. First, infection prevention must be strengthened and standardized in ICUs and high‐risk wards, with a focus on device‐associated infections and environmental decontamination. Second, antimicrobial stewardship programs should be expanded with real‐time resistance tracking, and empirical use of carbapenems should be restricted to slow further resistance selection. Third, investment in rapid diagnostics and novel therapeutic agents active against carbapenem‐resistant *A. baumannii* is urgently needed, alongside provincial‐level surveillance to identify local resistance hotspots. Finally, AMR should be prioritized in national health budgeting, with dedicated funding for stewardship training, laboratory capacity, and outbreak response.

Unless urgently addressed, the dual pressures of resistance and demographic change will continue to drive mortality and healthcare costs upward. Future studies should quantify the impact of specific interventions, such as screening protocols or stewardship initiatives, on both clinical outcomes and economic burden to guide evidence‐based policy in Iran and similar settings.

## Author Contributions

Conceptualization (visualization): Mohammadreza Salehi and Maryam Shafaati; screening (data curation, formal analysis, investigation, methodology, and resources): Sina Golestani, Ali Golestani, and Samaneh Akbarpour; risk of bias, data extraction, and validation: Sina Golestani, Ali Golestani, Samaneh Akbarpour, Mohammadreza Salehi, Maryam Shafaati, Arash Seifi, and Keyhan Mohammadi; data synthesis and software: Sina Golestani, Ali Golestani, and Arash Seifi; writing–original draft preparation: Sina Golestani and Ali Golestani; writing–review and editing: all authors; supervision: Mohammadreza Salehi, Maryam Shafaati, and Ali Golestani; project administration: Mohammadreza Salehi and Ali Golestani; and reviewing and editing: Mohammadreza Salehi, Maryam Shafaati, Samaneh Akbarpour, Keyhan Mohammadi, and Arash Seifi.

## Funding

The Research Centre for Antibiotic Stewardship and Antimicrobial Resistance (RCASAR), Tehran University of Medical Sciences (TUMS), provided funding for the study under grant number 73294.

## Disclosure

All authors have read and agreed to the published version of the manuscript.

## Ethics Statement

This research was conducted in accordance with the principles outlined in the Declaration of Helsinki. The findings are derived from estimates provided by the ARM 2021 study and comply with applicable guidelines and regulations. This study was approved by the research ethics committees of the Institute of Pharmaceutical Sciences at Tehran University of Medical Sciences (IR.TUMS.TIPS.REC.1403.115).

## Conflicts of Interest

The authors declare no conflicts of interest.

## Supporting Information

Additional supporting information can be found online in the Supporting Information section.

## Supporting information


**Supporting Information** Supporting 1. Figure S1. Trends of Associated and Susceptible Numbers and Rates of Burden and Mortality Caused by *Acinetobacter baumannii* in Iran from 2000 to 2021. Supporting 2. Figure S2. The Associated and Attributable Numbers and Rates of Burden and Mortality of *Acinetobacter baumannii* Infection Syndromes in Iran, in 2000 and 2021. Supporting 3. Figure S3. Economic Burden of *Acinetobacter baumannii* in Iran (2000–2021).

## Data Availability

The data that support the findings of this study are available from the corresponding author upon reasonable request.
