# Supplementary figures and images for: Burden and Economic Impact of Antimicrobial Resistance in Acinetobacter baumannii in Iran: A 2000–2021 Analysis
Source: Can J Infect Dis Med Microbiol. 2026 Aug 2;2026:6786134. doi: 10.1155/cjid/6786134 (PMC13430055; doi:10.1155/cjid/6786134)

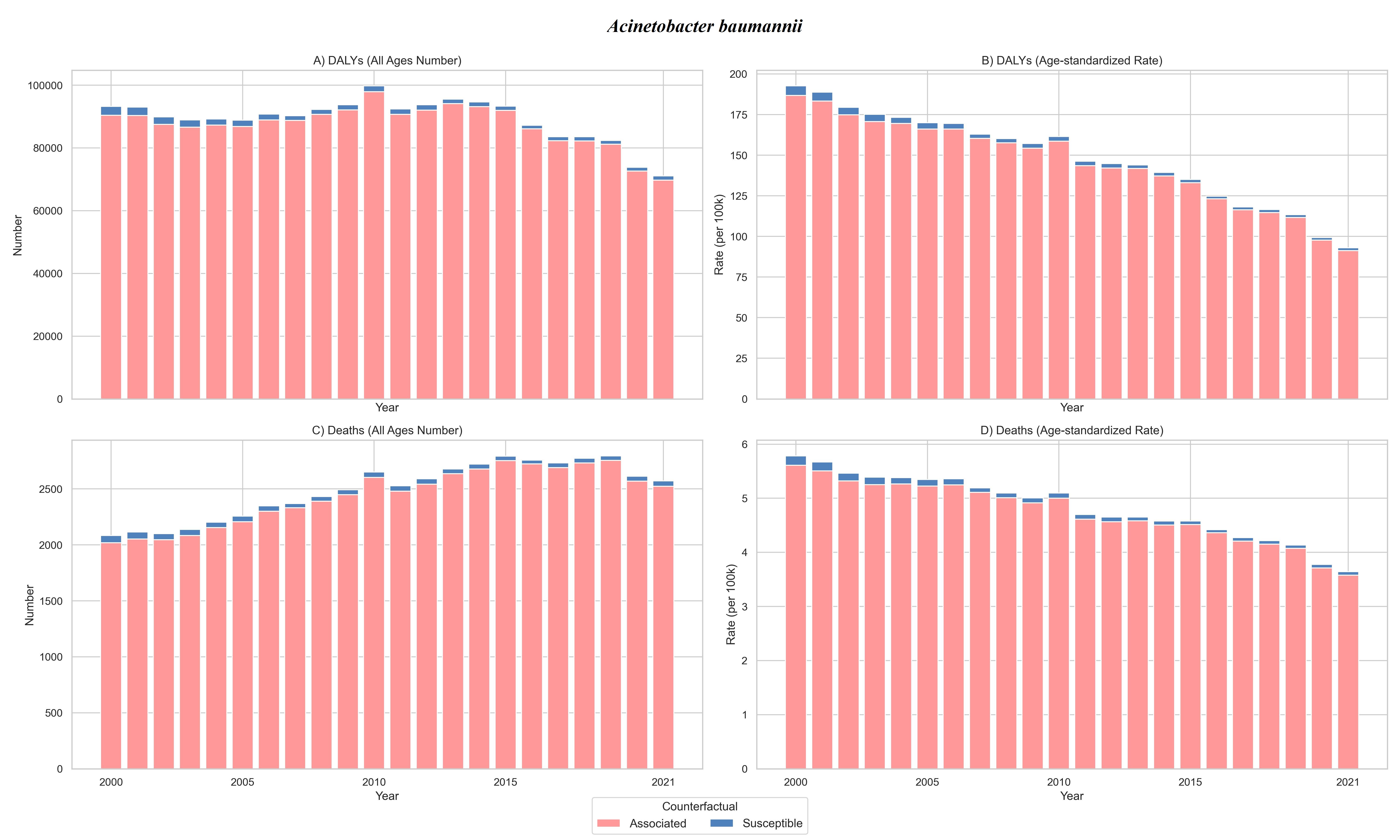

Supplement: Supplementary file 1 — Supporting Information Supporting 1. Figure S1. Trends of Associated and Susceptible Numbers and Rates of Burden and Mortality Caused by Acinetobacter baumannii in Iran from 2000 to 2021. Supporting 2. Figure S2. The Associated and Attributable Numbers and Rates of Burden and Mortality of Acinetobacter baumannii Infection Syndromes in Iran, in 2000 and 2021. Supporting 3. Figure S3. Economic Burden of Acinetobacter baumannii in Iran (2000–2021). [file CJID-2026-6786134-s001.zip › Supplementary Fig 1.jpg]

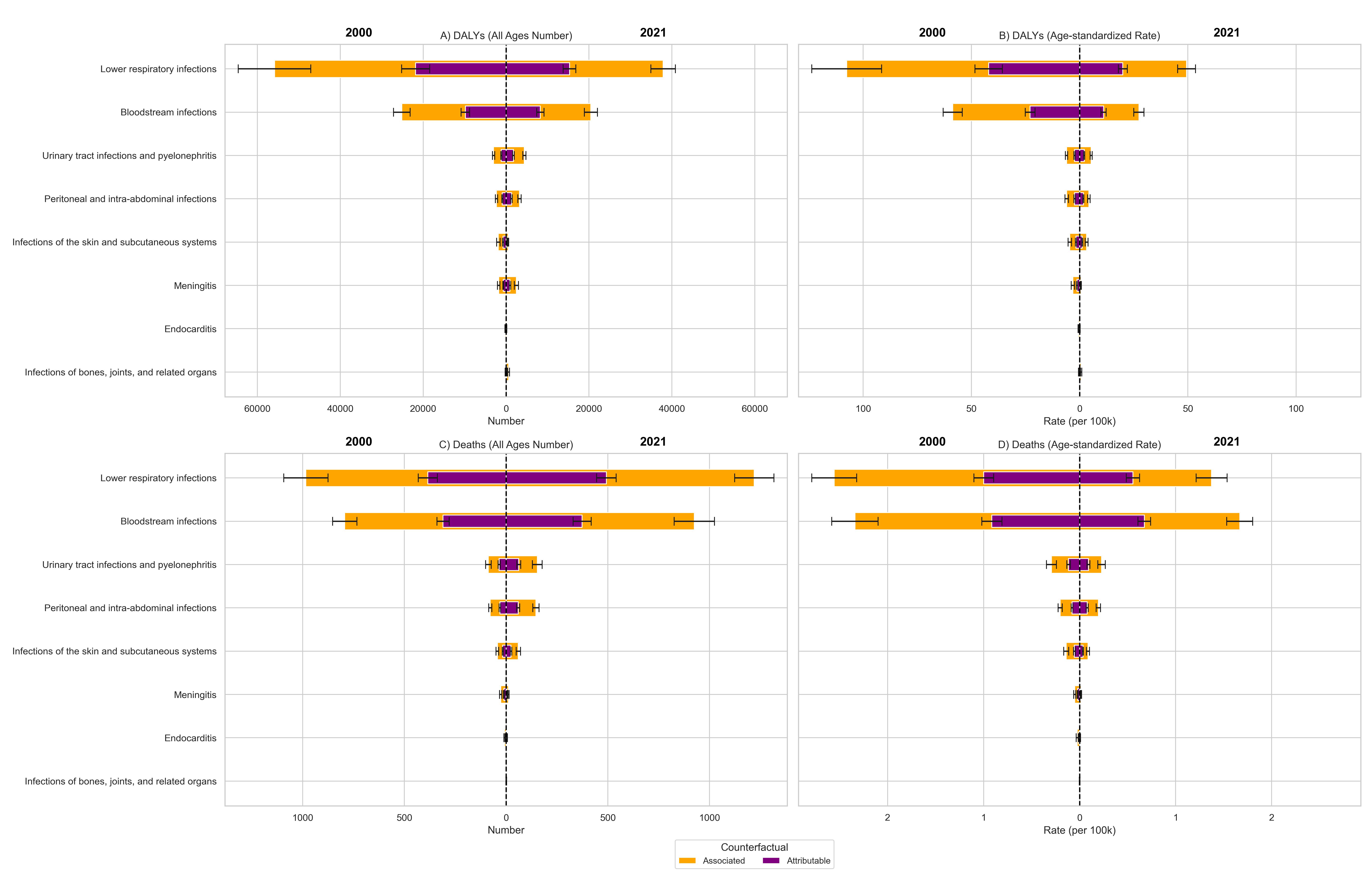

Supplement: Supplementary file 1 — Supporting Information Supporting 1. Figure S1. Trends of Associated and Susceptible Numbers and Rates of Burden and Mortality Caused by Acinetobacter baumannii in Iran from 2000 to 2021. Supporting 2. Figure S2. The Associated and Attributable Numbers and Rates of Burden and Mortality of Acinetobacter baumannii Infection Syndromes in Iran, in 2000 and 2021. Supporting 3. Figure S3. Economic Burden of Acinetobacter baumannii in Iran (2000–2021). [file CJID-2026-6786134-s001.zip › Supplementary Fig 2.jpg]

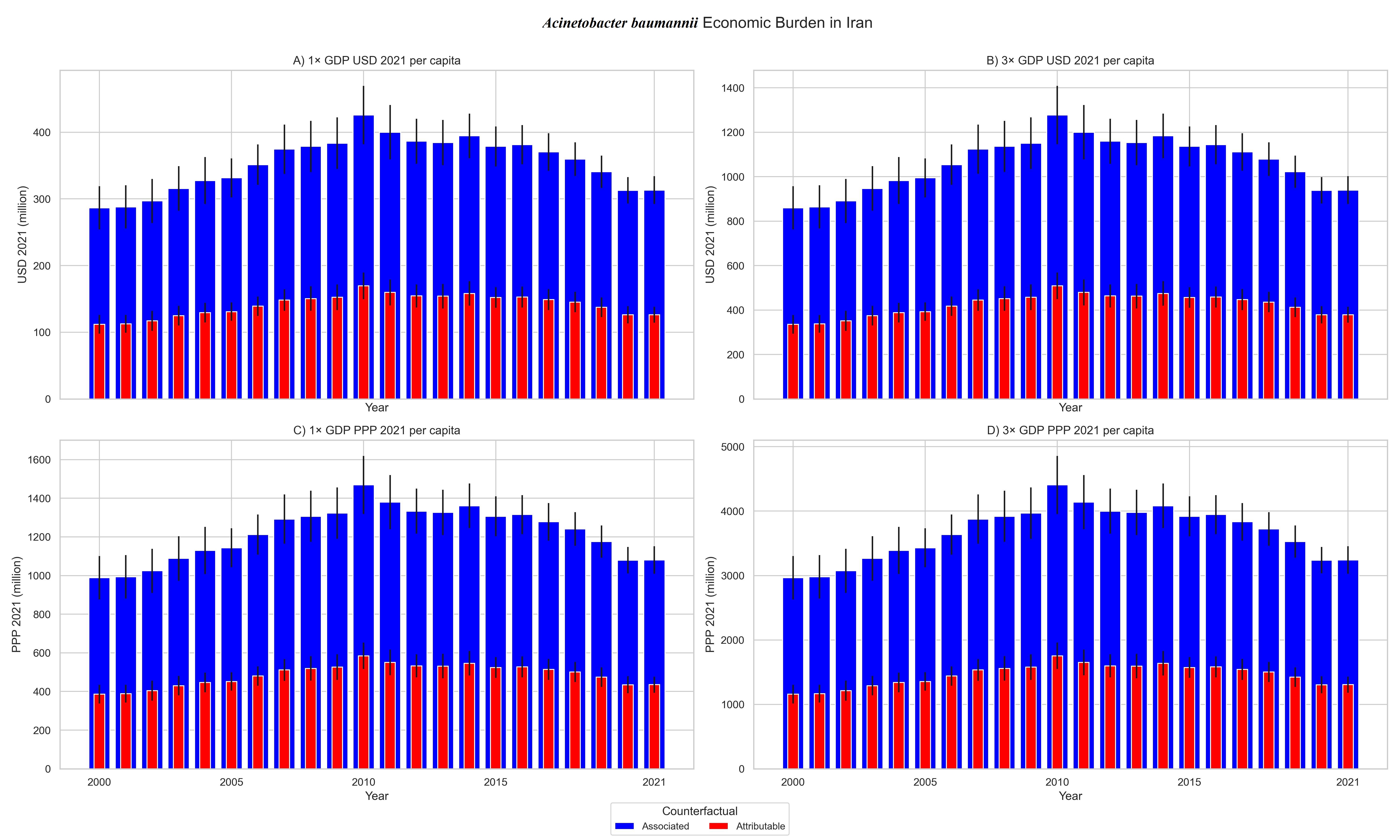

Supplement: Supplementary file 1 — Supporting Information Supporting 1. Figure S1. Trends of Associated and Susceptible Numbers and Rates of Burden and Mortality Caused by Acinetobacter baumannii in Iran from 2000 to 2021. Supporting 2. Figure S2. The Associated and Attributable Numbers and Rates of Burden and Mortality of Acinetobacter baumannii Infection Syndromes in Iran, in 2000 and 2021. Supporting 3. Figure S3. Economic Burden of Acinetobacter baumannii in Iran (2000–2021). [file CJID-2026-6786134-s001.zip › Supplementary Fig 3.jpg]
